# Chronic Aerobic Exercise Associated to Dietary Modification Improve Endothelial Function and eNOS Expression in High Fat Fed Hamsters

**DOI:** 10.1371/journal.pone.0102554

**Published:** 2014-07-18

**Authors:** Beatriz C. S. Boa, Maria das Graças C. Souza, Richard D. Leite, Simone V. da Silva, Thereza Christina Barja-Fidalgo, Luiz Guilherme Kraemer-Aguiar, Eliete Bouskela

**Affiliations:** 1 Laboratory for Clinical and Experimental Research on Vascular Biology (BioVasc), Biomedical Center, State University of Rio de Janeiro - Rio de Janeiro, RJ – Brazil; 2 Department of Cell Biology, Institute of Biology Roberto Alcântara Gomes, Biomedical Center, State University of Rio de Janeiro - Rio de Janeiro, RJ – Brazil; 3 Department of Internal Medicine, Faculty of Medical Sciences, Biomedical Center, State University of Rio de Janeiro – Rio de Janeiro, RJ – Brazil; Universidade Federal do Rio de Janeiro (UFRJ), Brazil

## Abstract

Obesity is epidemic in the western world and central adipose tissue deposition points to increased cardiovascular morbidity and mortality, independently of any association between obesity and other cardiovascular risk factors. Physical exercise has been used as non-pharmacological treatment to significantly reverse/attenuate obesity comorbidities. In this study we have investigated effects of exercise and/or dietary modification on microcirculatory function, body composition, serum glucose, iNOS and eNOS expression on 120 male hamsters treated for 12 weeks with high fat chow (HF, n = 30) starting on the 21^st^ day of birth. From week 12 to 20, animals were randomly separated in HF (no treatment change), return to standard chow (HFSC, n = 30), high fat chow associated to an aerobic exercise training program (AET) (HFEX, n = 30) and return to standard chow+AET (HFSCEX, n = 30). Microvascular reactivity in response to acetylcholine and sodium nitroprusside and macromolecular permeability increase induced by 30 minutes ischemia followed by reperfusion were assessed on the cheek pouch preparation. Total body fat and aorta eNOS and iNOS expression by immunoblotting assay were evaluated on the experimental day. Compared to HFSC and HFSCEX groups, HF and HFEX ones presented increased visceral fat [(mean±SEM) (HF)4.9±1.5 g and (HFEX)4.7±0.9 g vs. (HFSC)*3.0±0.7 g and (HFSCEX)*1.9±0.4 g/100 g BW]; impaired endothelial-dependent vasodilatation [Ach 10^−8^ M (HF)87.9±2.7%; (HFSC)*116.7±5.9%; (HFEX)*109.1±4.6%; (HFSCEX)*105±2.8%; Ach10^−6^ M (HF)95.3±3.1%; (HFSC)*126±6.2%; (HFEX)*122.5±2.8%; (HFSCEX)*118.1±4.3% and Ach10^−4^ M (HF)109.5±4.8%; (HFSC)*149.6±6.6%; (HFEX)*143.5±5.4% and (HFSCEX)*139.4±5.2%], macromolecular permeability increase after ischemia/reperfusion [(HF)40.5±4.2; (HFSC)*19.0±1.6; (HFEX)*18.6±2.1 and (HFSCEX)* 21.5±3.7 leaks/cm^2^), decreased eNOS expression, increased leptin and glycaemic levels. Endothelial-independent microvascular reactivity was similar between groups, suggesting that only endothelial damage had occurred. Our results indicate that an aerobic routine and/or dietary modification may cause significant improvements to high fat fed animals, diminishing visceral depots, increasing eNOS expression and reducing microcirculatory dysfunction.

## Introduction

Life style changes seem to mark contemporary men, from active to sedentary and from healthy alimentation to high fat/salt one. With that, now is clear that the adipose tissue represents much more than simply an energy storage site, being also a powerful endocrine organ with numberless functions. The Western profile of high fat feeding is directly related to adipocyte hypertrophy, hypoxia and, as consequence, its dysfunction comprising anatomic and functional disturbances due to prolonged positive caloric balance in genetic susceptible individuals [1]. Consequences of adipose tissue dysfunction involve systemic effects such as micro/macrovascular dysfunction, type 2 diabetes mellitus (T2DM), hypertension and cardiovascular diseases (CVD). In obesity, there is an increased production of free fatty acids, angiotensinogen, leptin, resistin and inflammatory mediators that might be involved in mechanisms of obesity-associated microvascular dysfunction [2]. In fact, visceral and truncal subcutaneous adipose tissues are associated to microvascular dysfunction even in lean subjects [3] and weight loss improves endothelial function and decreases inflammation in an obese population [4].

Endothelial dysfunction (ED), reduction of bioavailability of vasodilators, mainly NO, may also be characterized by a state of endothelial activation where pro-inflammatory, proliferative and pro-coagulant milieu predominates [5]. Therewith, ED is a clinical syndrome able to predict and also be associated to cardiovascular events [6]. Indeed, the immense endothelium surface area in the microcirculation compared to conductance vessels makes this region more vulnerable to effects of its dysfunction. Therefore, microvascular dysfunction emerges as an independent predictor of cardiovascular risk and systemic inflammation [7] and it has been associated to hypertension [8], presence of chest pain in absence of coronary artery disease [9] and classical cardiovascular risk markers [6]. The cheek pouch, an invagination of the oral mucosa that extends under the subcutaneous tissue down to the shoulder region, is an appropriate preparation to study microcirculatory function/dysfunction due to its clarity and stability.

Physical activity has gained visibility since it is seen as non- pharmacological treatment of excessive weight and adiposity as well as its co-morbidities, and far more accessible to several patient populations. The skeletal muscle constitutes approximately 40% of total body weight, responsible for 30% of energy expenditure and considered the most important determinant of peripheral vascular sensibility to insulin [10]. It is also an important place for uptake, storage and liberation of glucose [11]. Regular exercise practice reduces primary [12] and secondary [13] vascular events. Therewith, it is the best non-pharmacological treatment for the vasculature inasmuch it exerts several of its physiological improvements through modification of laminar shear stress, as a result increased eNOS expression (endothelium nitric oxide synthase), arterial stiffness and decreased oxygen reactive species (ROS) production [14]–[15]. These effects reflect a direct interaction between exercise's actions and the microcirculation.

Both exercise and dietary modifications culminate in changes of the energetic balance, which modify adiposity and improve metabolic parameters [15]–[17], but data in experimental literature concerning microcirculatory analysis when these two treatments are combined are scarce. In this study we have investigated effects of an aerobic exercise training (AET) associated or not to dietary modification on microcirculatory function, body composition, serum glucose, iNOS and eNOS expression on male hamsters to evaluate if these non-pharmacological treatments, namely exercise and diet, could attenuate/reverse the consequences of high fat feeding.

## Experimental Methods

**Figure 1:**
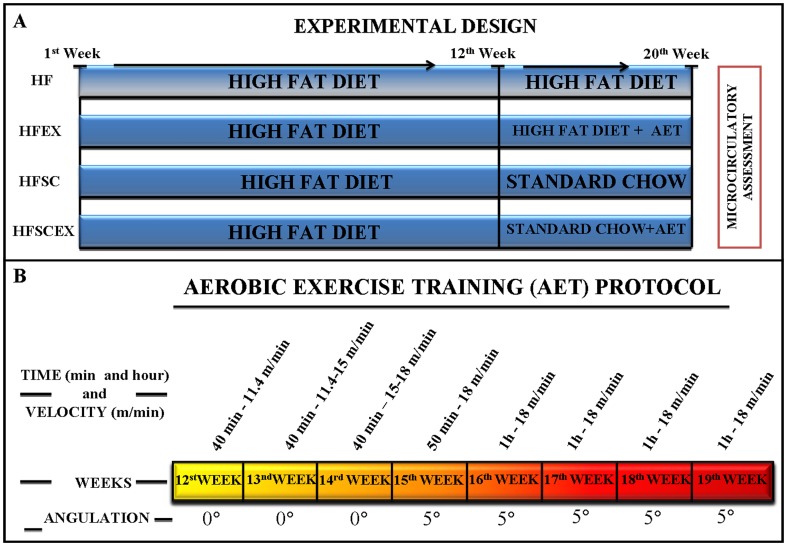
Experimental design and Aerobic Exercise Training. (A) Experimental design with dietary modification and aerobic exercise training, after week 12. (B) Aerobic Exercise Training (AET) performed by hamsters, organized by weeks, time, speed and angulations. High fat fed hamsters were subjected to AET in the last two months associated or not to diet modification.

Experiments were performed on male Syrian golden hamsters (n = 120; *Mesocricetus auratus*, ANILAB, Paulínea, SP, Brazil), acclimatized at 20±1°C, with 12 hours cycles of day/night controlled by timer and light from 6:00AM to 18:00PM. On the 21th day after birth, hamsters started to be feeding with high fat (HF) chow [18] for twelve weeks. From the twelfth to the twentieth week, animals were randomly divided into four different groups: HF (20 weeks, fed with high fat chow), HF + Standard Chow (HFSC – from 12 to 20^th^ week animals returned to standard chow), HF + Exercise (HFEX – similar to HF associated to an exercise routine in the last eight weeks) and HF+SC/Exercise (HFSCEX – similar to HFSC, associated to an exercise routine in the last eight weeks). Nutritional characteristics of used high fat chow may be seen on table 1, in detail. The experimental design and the aerobic exercise training protocol [19] can be seen on figure 1. Animals had unrestricted access to food and water.

**Table 1:** Nutritional composition and comparison between experimental chows.

	Standard Chow (%)	High Fat Chow (%)
**Standard Chow** a	100	60
**Hydrogenated Vegetable Fat** b	-	25
**Condensed Milk** c	-	15
***Macronutrients***		
**Protein**	23	12
**Fat**	6	50
**Carbohydrates**	71	38
***Total Energy***		
**KJ/Kg**	17.9	23

a Standard chow for rodents; Nuvital, Nuvilab - Colombro Paraná - Brazil;

b Shortening Primor: Gaspar, Santa Catarina - Brazil; c Condensed Milk Moça Nestlé, São Paulo – Brazil.

c Condensed Milk Moça Nestlé, São Paulo – Brazil.

On the day of experiment, the right femoral vein and the left femoral artery were cannulated (0.28 mm internal/0.61 mm outer diameters) for drug injection and mean arterial pressure (MAP) and heart rate (HR) measurements (Biopac, Santa Barbara, CA, USA, Spectramed pressure transducer). A tracheal tube was inserted to facilitate spontaneous breathing (room air). The cheek pouch was gently everted and mounted on an experimental chamber as previously described [20]. All preparations were superfused at a rate of 4.0 ml/min by a HEPES-supported HCO_3_^−^-saline solution [composition in mM: NaCl 110.0, KCl 4.7, CaCl_2_ 2.0, MgSO_4_ 1.2, NaHCO_3_ 18.0, N-2-hydroxyethylpiperazine-N′-2- ethanesulfonic acid (HEPES) 15.39 and HEPES Na^+^-salt 14.61] bubbled with 5% CO_2_–95% N_2_, to maintain the superfusion solution with pH at 7.4 and the temperature at 36.5°C. Preparations were placed under an intravital microscope (Leica DMLFS, Wetzlar, Germany, magnification x600, NA 0.65) coupled to a closed-circuit TV system and allowed to rest for 30 min before measurements were taken. Images were recorded in sVHS and analyzed after the experiment.

### Ethics Statement

The protocol was approved by the Ethical Committee of the State University of Rio de Janeiro (CEUA/061/2010). The investigation was strictly conducted according to the *Guide for Care and Use of Laboratory Animals* published by the US National Institute of Health (NIH Publication No. 85–23, revised in 1996). Animals were euthanized through cardiac puncture, under anesthesia, for blood analysis. All efforts were made to minimize their suffering.

All surgical procedures were performed under anesthesia induced by an intraperitoneal injection of 0.1–0.2 ml of sodium pentobarbital (Pentobarbital sodique, 60 mg/ml, Sanofi Santé Animale, Paris, France) and maintained with α-chloralose [100 mg/kg body weight (Sigma Chemicals, St. Louis MO, USA)] given. Throughout surgery and experiment, hamsters were placed on a heating pad, controlled by a rectal thermistor, in order to maintain their body temperature at 36.5°C (LTB 750 Thermostat System, Uppsala, Sweden).

### Microvascular research model

The hamster cheek pouch is an appropriate preparation to study effects of dietary change associated to aerobic exercise. This preparation consists on an invagination of the oral mucosa under the subcutaneous tissue down to the shoulder region. Its blood supply comes mainly from the carotid arteries, although some blood is diverged to the retractor muscle, also part of the cheek pouch structure. The preparation remains stable for 5–6 h for arteriolar reactivity to acetylcholine (ACh) [21], for macromolecular permeability increase induced by bradykinin [22] and for spontaneous arteriolar vasomotion [20]. There are several advantages related to the use of this preparation: 1) ease and relatively non-traumatic access make it useful in studies requiring repeated observations of the same site; 2) highly vascularized with all classes of microcirculatory vessels visible within the microscopic field, allowing observation and comparison of various segments; 3) clarity and optical properties are good when compared to other densely vascularized tissues; 4) presence of skeletal muscle and cutaneous microcirculatory beds [21].

### Microvascular reactivity and macromolecular permeability

Three 2^nd^ to 3^rd^ order arterioles were selected and studied in each preparation, taking into account its position, in order to return exactly to the same sites for consecutive evaluations after topical application of vasoactive drugs (e.g. presence of fat cells and bifurcations). As a precaution, a conversion ruler (mm to µm) was used to guarantee that recorded vessels encompassed 2^nd^ to 3^rd^ order and mean luminal diameter of 100 µm was considered as upper limit. Such choices were important to keep homogeneity of microvessels' response after topical application of vasoactive agents (e.g. acetylcholine). Mean internal microvessel diameter was determined using an Image Shearing device (Vista Electronics, model 908, San Diego, CA, USA), at baseline and after topical application, 10 min each, of three concentrations of either acetylcholine [Ach 10^−8^, Ach 10^−6^ and Ach 10^−4^ M (Sigma Chemicals, St. Louis, MO, USA)] or sodium nitroprusside [SNP 10^−8^, SNP 10^−6^ and SNP 10^−4^ M (Sigma Chemicals, St. Louis, MO, USA)] in a cumulative dose-response curve.

Induction of ischemia/reperfusion-induced changes was made with an air inflatable tourniquet placed distally of the preparation around the neck of the pouch (HCP) and inflated for 30 min to suppress local blood flow as previously described, Persson et al [23]. FITC-dextran (150 kDa, TdB Consultancy, Uppsala, Sweden) was injected i.v. 25 mg/100 g body weight for measurements of macromolecular permeability changes. FITC-dextran (150 kDa, 25 mg/100 g body weight, 5% solution, TdB Consultancy, Uppsala - Sweden). Permeability for large molecules was quantified by counting the number of leaky sites (leaks) in the prepared area (1 cm^2^) using an UV-light microscope (40× magnification). Leaks are defined as visible extravascular spots (diameter>100 µm) of FITC-dextran in post-capillary venules seen under fluorescent light. The number of leaks was counted at baseline, during reperfusion and after 30 min ischemia in times 0, 5 and 10 min (T0, immediately after; T_5_; 5 min after and T_10_; 10 min after tourniquet release). Preparations presenting more than 10 spontaneous leaks or petechiae at baseline were excluded due to previous damage to the tissue.

### Adipose tissue collection

Urogenital, mesenteric and retroperitoneal fat depots were collected according to Cinti [24], weighed and store at −80°C.

### Blood samples analysis

Blood samples were collected before the experimental protocol from the saphenous vein, using a capillary tube containing heparin (Heparina Perfecta, 75 mm length – São Paulo, SP - Brazil) for glucose analysis (One touch ultra - Johnson e Johnson, Medical Brazil) [25]. Immediately after the sacrifice of the animal, blood was collected by cardiac puncture, centrifuged [3000 r min−1 (1157×g) for 10 min at 4°C] and stored at −80°C for total triglycerides (TG), total cholesterol (TC), and high-density lipoprotein (HDL) determinations using the Biosystem kit with sensitivity of 0.7 mMol l−1, 0.14 mMol l−1, and 0.5 mMol l−1, respectively. Low-density lipoprotein (LDL) = TC−HDL−(TGL×0.2) and very low density lipoprotein (VLDL) = TGL/5) were calculated by Friedwald's equation [26].

### Preparation of the aortic extract

After the experimental protocol and microvascular study, the thoracic aorta was dissected from six animals per group, excised and its strips lysed in 50 mM HEPES (pH 6.4), 1 mM MgCl_2_, 10 mM EDTA, 1% Triton X-100, 1 mg/ml DNase, 0.5 mg/ml RNase containing the following protease inhibitors: 1 mM PMSF, 1 mM benzamidine, 1 mM leupeptin, and 1 mM soybean trypsin inhibitor (Sigma Chemicals, St. Louis, MO, USA).

### Immunoblotting essay

Total protein content of aortic extracts was determined by Bradford's method [27]. Samples (30 µg in total) were resolved by 10% SDS-PAGE and proteins were transferred to PVDF membranes (Hybond- P, Amersham Pharmacia Biotech, San Francisco, CA, USA). Rainbow markers (Amersham Pharmacia Biotech, San Francisco, CA, USA) run in parallel to estimate molecular weights. Membranes were blocked with Tween-PBS (0.1% Tween-20 in PBS) containing 5% bovine serum albumin and incubated with specific primary antibody: anti-iNOS (1∶500; Santa Cruz Biotechnology, Santa Cruz, CA, USA) and anti-eNOS (1∶500; Santa Cruz Biotechnology, Santa Cruz, CA, USA). After extensive washings in Tween-PBS, PVDF sheets were incubated with appropriate secondary biotin-conjugated antibody conjugated to biotin (Santa Cruz Biotechnology, Santa Cruz, CA, USA) for 1 h and then incubated with horseradish peroxidase conjugated to streptavidin (1∶10000; Caltag Laboratories, Burlingame, CA, USA). Immunoreactive proteins were visualized using ECL system (Pierce ECL Chemiluminescent Substrate; Thermo Scientific, Rockford USA). Membranes were stripped with buffer containing 62.5 mM Tris–HCl, 2% SDS and 100 mM β-mercaptoethanol and re-probed similarly with anti-ERK2 antibody (1∶1000; Santa Cruz Biotechnology, Santa Cruz, CA, USA). Films were scanned and analyzed semi quantitatively and iNOS and eNOS were normalized to actin levels. Bands were quantified by densitometry, using Image J 1.34 s Software (NIH, USA).

### Exercise protocol

After 12 weeks of HF feeding, hamsters (5 to 6 months of age) were randomly assigned to an exercise practice (n = 60). One week before the beginning of the exercise protocol, they were placed in the treadmill machine at lower speed for a short time for adaptation. The exercise training consisted of 8 weeks of running on a motor treadmill (INSIGHT – EP 131 – Ribeirão Preto, SP, Brazil), 5 times/week, raising weekly time and speed until the limit of 60 min at 18 m/min (Figure 1) [19]. Exercise was conducted at 50–70% of VO_2_ maximum, featuring the overall AET protocol as of moderate intensity. Measures of RQ (respiratory quotient) were set at 1 to designate respiratory exhaustion and conducted in 0.89, characterizing the exercise as aerobic. Animals did not pass through exercise training 24–36 h before the experimental day.

### Leptin evaluation

Leptin levels were evaluated in blood serum obtained by heart puncture (centrifugation −2.500 rpm for 12 minutes – Eppendorf, Centrifuge 5804R,Hamburg – Germany) using an ELISA kit (Peprotech ELISA Development Kit, 900-K76 Lot# 0608076 – London, UK). The assay sensitivity was 20 pg/ml. Inter and intra-assay coefficient of variation were, respectively, 6.4% and 5.1%, Samples were assayed in triplicate.

### Data analysis

Statistical analysis was performed initially using Kolmogorov-Smirnov normality test and homoscedasticity tests (Bartlett criterion). Body mass, fat percentage, fat depots, lipid profile and blood measures presented normal distribution and are reported as means±standard error of the mean (S.E.M.). Microvascular reactivity and macromolecular permeability did not show normal distribution and are reported as median [10–90 percentiles]. Microvessel diameters are presented as relative changes, normalized to baseline. Microcirculatory data between groups were analyzed using Kruskal-Wallis test, followed by post hoc Dunn test. Group differences in arteriolar diameter responses to Ach were determined by Friedman's test. Variables with normal distribution were analyzed using One Way ANOVA to compare differences between groups, followed by post hoc Tukey test. Statistical analysis was performed using Prism 5.01 (Graphpad Inc., San Diego, CA, USA). Results were considered statistically different when p<0.05.

## Results

One hundred and twenty male hamsters used were randomly distributed into four experimental groups: high fat (HF, n = 30), high fat + Exercise (HFEX, n = 30), high fat +Standard chow (HFSC, n = 30), high fat + Standard chow + exercise (HFSCEX, n = 30). Most data are shown as mean±SEM, except for microcirculatory assessments presented as median (10–90 percentiles).

**Figure 2:**
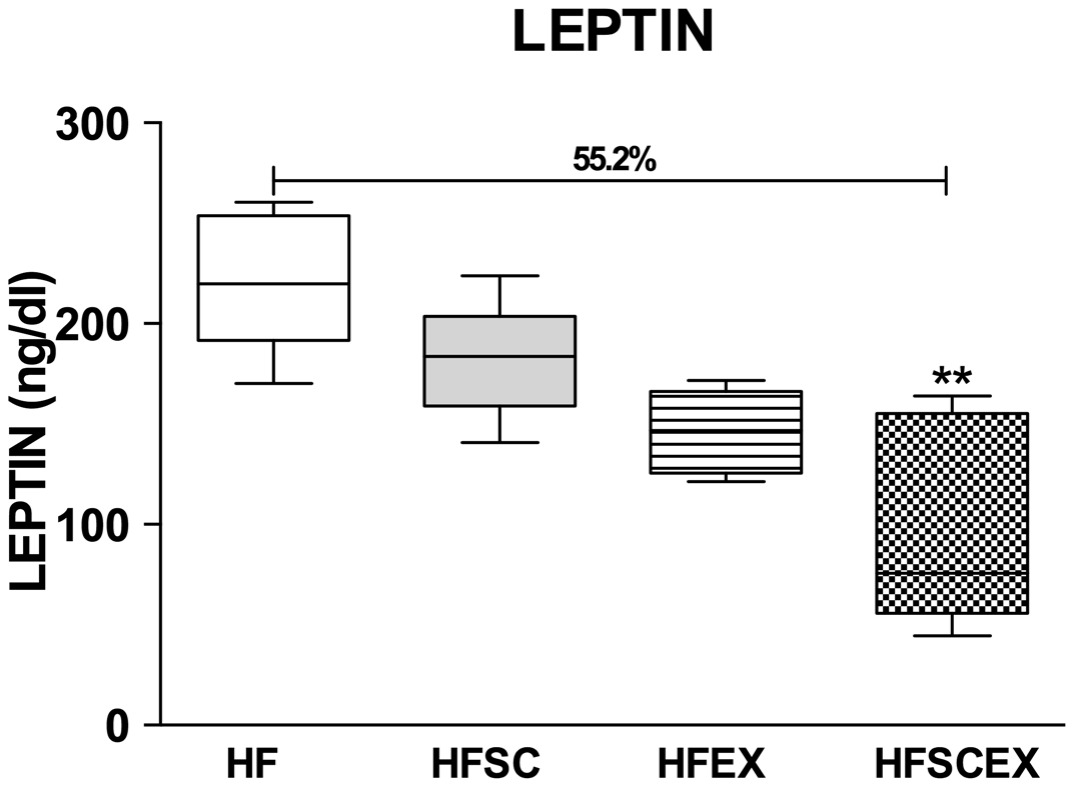
Leptin evaluation. Data are presented as mean±standard error of the mean HF =  High Fat Diet. HFSC =  High Fat Diet + Standard Chow. HFEX =  High Fat Diet + Exercise. HFSCEX =  High Fat Diet +Standard Chow/Exercise. *Significantly different from the HF group (p<0.01).

Fat overload elicits significant increase on body weight that could be altered after interventions (experimental design shown on figure 1). Treated groups (HFSC and HFSCEX) showed significant reduction not only on body weight [HFSC vs. HF and HFEX reduction of 10.24% and 9.56% respectively, HFSCEX vs. HF and HFEX reduction of 9.66% and 8.8% respectively; p<0.0001], but also on naso-anal length [HFSC vs. HF and HFEX reduction of 9.0% and 6.9% respectively, HFSCEX vs. HF and HFEX reduction of 6.1% and 2.9% respectively; p = 0.0011] and fat depots [fat% HFSC vs. HF and HFEX with differences of 29.7% and 32.3% respectively, HFSCEX vs. HF and HFEX with differences of 26.6% and 29.2% respectively, p<0.001; visceral HFSC vs. HF and HFEX with differences of 30% and 31.4% respectively, HFSCEX vs. HF and HFEX with differences of 28% and 29.4% respectively, p<0.0001]. Concerning hemodynamic responses, mean arterial pressure (MAP) and heart rate (HR) showed linear improvements on all treated groups (HFSC, HFEX and HFSCEX) {[MAP – HFEX vs. HF and HFSC with differences of 20.2% and 9.2% respectively; HFSCEX vs. HF and HFSC with differences of 31.1% and 21.7% respectively, p<0.001]; [HR - HFEX vs. HF and HFSC with differences of 8.9% and 11.6% respectively; HFSCEX vs. HF and HFSC with differences of 15% and 16.4% respectively p<0.01]}. These results are shown on table 2. In terms of blood analysis, no difference could be found between groups concerning total cholesterol, LDL, HDL and triglycerides, but fasting blood glucose began to show a tendency towards reduction on treated groups (HFEX and HFSCEX) as seen on table 3. HF animals had significantly higher levels of leptin (figure 2), that gradually decreased from HF to HFSCEX [(HF) 222±15.5 ng/dl, (HFSC) 181±13.1 ng/dl, (HFEX) 146.2±8.3 ng/dl and [(HFSCEX)*99.4±23.4 ng/dl; *significantly different from HF group; p = 0.0039].

**Table 2:** Assessments performed on experiment day.

	HF	HFSC	HFEX	HFSCEX
**Body mass (g)**	171.8±2.8	* +154.2±2.6	170.5±2.8	* +155.5±1.9
**Naso-anal length (cm)**	18±0.29	*16.2±0.32	17.4±0.18	*16.9±0.23
**Body Fat (%)**	15.8±0.6	* +11.1±0.33	16.4±0.53	* +11.6±0.28
**Uro Depot**	2.6±0.37	2.5±0.29	3.0±0.24	2.6±0.28
**Vis Depot**	5.0±0.79	* +3.5±0.39	5.1±0.54	* +3.6±0.52
**Ret depot**	1.5±0.23	1.1±0.11	1.4±0.11	1.2±0.11
**MAP**	159.5±3.4	*140.3±5.8	*127.3±5.7	# *109.8±5.9
**HR**	378.9±6.8	386±13.7	# *344±9	# *322.7±9.2

All values are presented as mean±standard error of the mean. HF =  High Fat diet. HFSC =  High Fat Diet + Standard Chow. HFEX =  High Fat Diet+Exercise. HFSCEX =  High Fat Diet +Standard Chow/Exercise (n = 10/per group). Fat (%)  =  Fat percentage; Uro depot =  Urogenital fat depot (g/100 g of body mass); Vis depot  =  Visceral fat depot (g/100 g of body mass); Ret depot  =  Retroperitoneal fat depot (g/100 g of body mass). MAP =  Mean arterial pressure. HR =  Heart rate.

*Significantly different from HF group (p<0.001).

+ Significantly different from the HFEX group (p<0.01).

# Significantly different from the HFSC group (p<0.01).

**Table 3:** Lipid profile and Glucose evaluation of high fat chow (HF), High fat+Standard chow (HFSC), High fat +Aerobic Exercise (HFEX) and High fat +Standard Chow/Exercise (HFSC/EX) groups.

	HF	HFSC	HFEX	HFSCEX
**TC (mmol/l)**	2.380	1.890	1.990	2.070
**HDL(mmol/l)**	1.580	1.345	1.500	1.505
**LDL(mmol/l)**	0.4950	0.3300	0.1850	0.1800
**VLDL(mmol/l)**	0.2800	0.3400	0.3050	0.3300
**TGL(mmol/l)**	1.415	1.718	1.515	1.650
**Glycemia (mg/dL)**	94.5±8.4	91±6.8	86.2±5.1	81.2±5.8

All values are presented as mean±S.E.M. No significant difference was found between groups. HF =  High Fat Diet. HFSC =  High Fat Diet +Standard Chow. HFEX =  High Fat Diet +Exercise. HFSCEX =  High Fat Diet +Standard Chow/Exercise (n = 10/per group). TC =  total cholesterol; HDL =  high density lipoprotein; LDL =  low density lipoprotein; VLDL =  very low density lipoprotein; TGL =  triglycerides.

**Figure 3:**
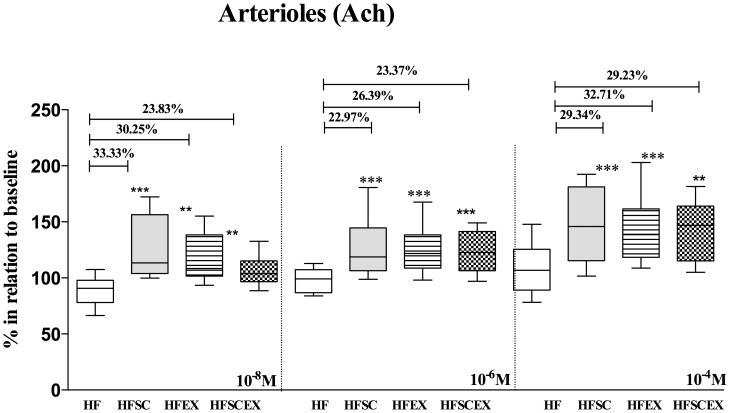
Mean arteriolar diameters after topical application of three concentrations of acetylcholine (10^−8^, 10^−6^ and 10^−4^ M) to the cheek pouch of hamsters fed with high fat diet (HF and HFEX, n = 10 each) and that had dietary modification associated or not to AET (HFSC and HFSCEX, n = 10 each) during 20 weeks. Data are shown as changes relative to baseline considered as 100%. Data are expressed as median±10–90 percentile, represented by vertical bars. *Significantly different from sedentary obese control group (p<0.001 and p<0.01, respectively) in all concentrations of Ach.

**Figure 4:**
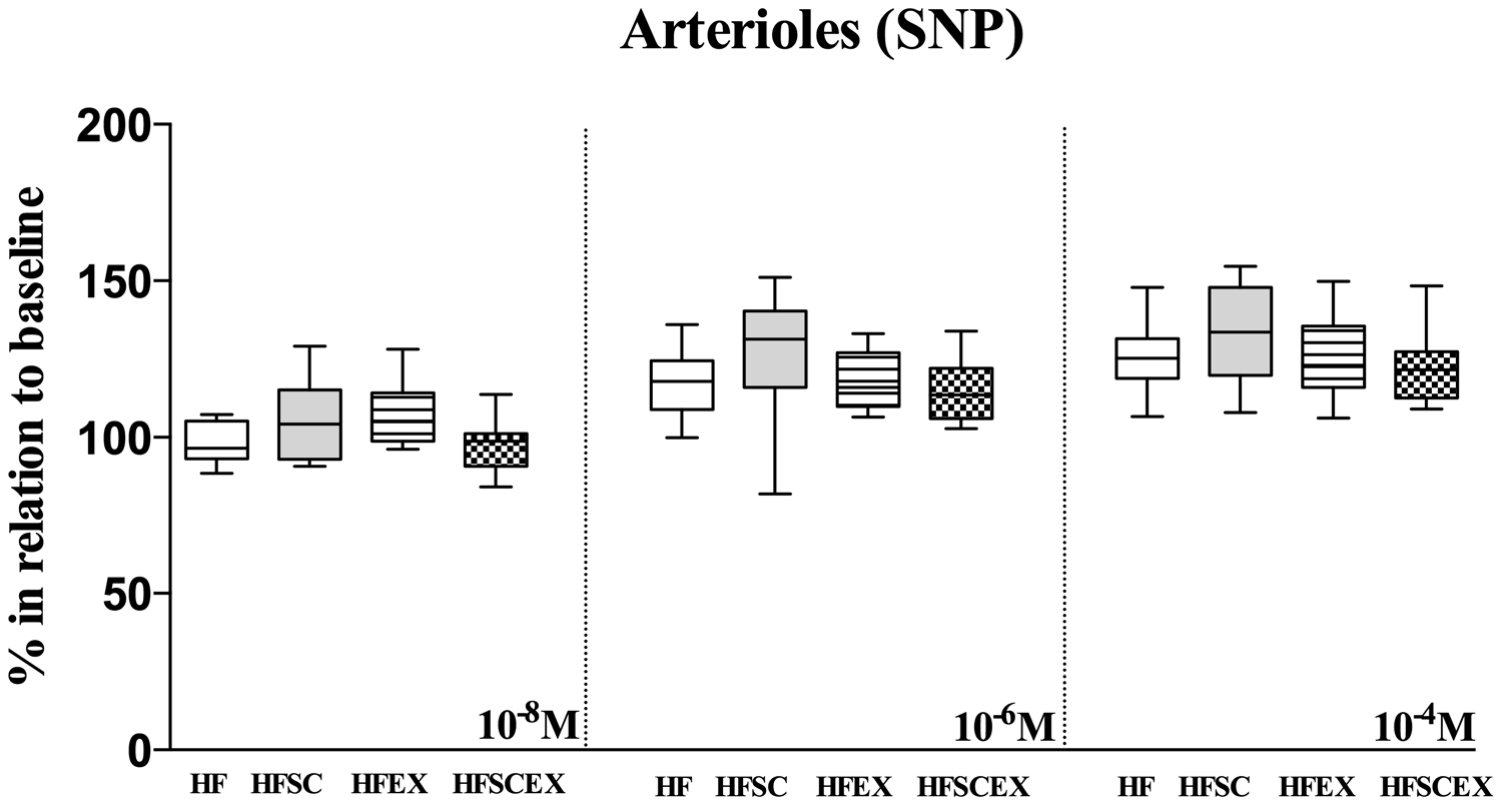
Mean arteriolar diameters after topical application of three concentrations of sodium nitroprusside (10^−8^, 10^−6^ and 10^−4^ M) to the cheek pouch of hamsters fed with high fat diet (HF and HFEX, n = 10 each) and that had dietary modification associated or not to AET (HFSC and HFSCEX, n = 10 each) during 20 weeks. Data are shown as changes relative to baseline considered as 100%. Data are expressed as median±10–90 percentile. No difference was found between groups for sodium nitroprusside. HF =  High Fat Diet. HFSC =  High Fat Diet + Standard Chow. HFEX =  High Fat Diet + Exercise. HFSCEX =  High Fat Diet +Standard Chow/Exercise.

The vasomotor activity was studied in more detail on two hundred and forty 2^nd^ to 3^rd^ order arterioles with baseline mean internal diameter ranging from 40 to 90 µm. No significant differences on baseline diameter were observed in any group and all of them presented robust blood flow and single flowing red blood cells were not discernible at any time. Changes in the microvasculature consisted on reverting most endothelial-dependent microvascular dysfunction demonstrated by Ach responses on arteriolar diameter on HFSC, HFEX and HFSCEX animals, while on HF ones it has been observed even a contractile phenotype. Data are shown as median (1st and 4th quartiles). This scenario was observed in all doses utilized, in values compared to baseline, considered as 100% [10^−8^ M - HF 90.70(78.00–97.80)%, HFSC *113.5(104.0–156.5)%, HFEX *108.3(101.6–138.3)%, HFSCEX *103.8(96.59–115.2)%; *significantly different from the HF group - p<0.0001]; [10^−6^ M - HF 99.04 (86.80–107.2)%, HFSC *118.7 (106.5–144.6)%, HFEX *121.7 (108.7–138.5)%, HFSCEX *122.8(106.4–141.5)%; *significantly different from the HF group - p<0.0001]; [10^−4^ M - HF 106.8(89.14–125.5)%, HFSC *145.9 (115.3–181.2)%, *HFEX 138.8(118.3–161.6)%, *HFSCEX 147.0(115.2–164.1)%; *significantly different from the HF group - p<0.0001] (Figure 3). No significant differences between groups could be observed with topical application of sodium nitroprusside, as seen on figure 4.

**Figure 5:**
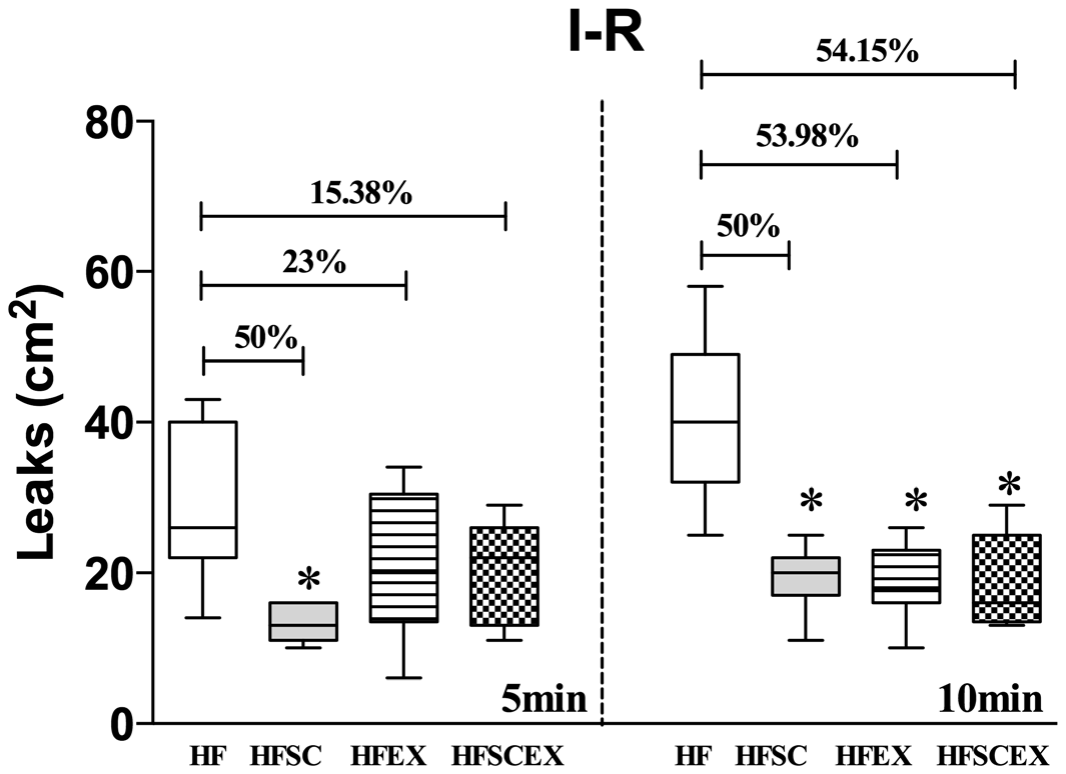
Macromolecular permeability at post capillary venules after 30 minutes ischemia of the cheek pouch of hamsters fed with high fat chow (HF and HFEX, n = 10 each) and with dietary modification associated or not to AET (HFSC and HFSCEX, n = 10 each) during 20 weeks. T5, 5 minutes after and T10, 10 minutes after tourniquet release. Data are expressed as median ± 10–90 percentile, represented by vertical bars. * Significantly different from the HF group (p<0.05). HF =  High Fat Diet. HFSC =  High Fat Diet + Standard Chow. HFEX =  High Fat Diet + Exercise. HFSCEX =  High Fat Diet +Standard Chow/Exercise.

Macromolecular permeability after ischemia/reperfusion procedure, measured at 10 minutes after the onset of reperfusion, was significantly higher on HF animals and, after all intervention protocols (HFSC, HFEX and HFSCEX groups), animals showed significantly lower number of leaks at 10 minutes [HF 40.5(32–49)leaks/cm^2^, HFSC *19.0 (17–22)leaks/cm^2^, HFEX *18.6(16–23)leaks/cm^2^and HFSCEX *16.0(13.5–25)leaks/cm^2^, *Significantly different from the HF group; p<0.0057 - as visualized on figure 5].

**Figure 6:**
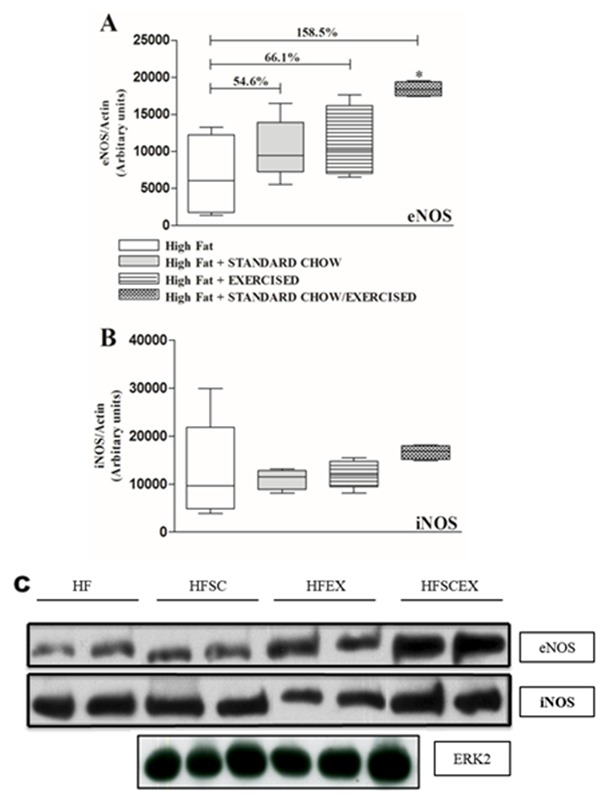
Western blot essays of eNOS (A and B) and iNOS (A and C) in the thoracic aorta of hamsters fed with high fat (HFD) or standard diet (Control) during 16 weeks. Data are expressed as means±S.E.M represented by vertical bars. *Significantly different from the control group (p<0.05). HF =  High Fat Diet. HFSC =  High Fat Diet + Standard Chow. HFEX =  Obese + Exercise. HFSCEX =  High Fat Diet +Standard Chow/Exercise.

eNOS expression assay showed a linear increase in all treated groups, but significant differences compared to HF were only found on HFSCEX (p = 0.032- figure 6). No significant differences were found on iNOS evaluations.

## Discussion

Our initial hypothesis was confirmed since microcirculatory dysfunction induced by fat overload was significantly attenuated by chronic moderate aerobic exercise training program (AET) associated or not to dietary modification. This study represents a continuation of our previous work [28] in which 16 weeks HF feeding was responsible for inciting visceral obesity, insulin resistance and microvascular damage.

In order to evaluate AET's and dietary modification effects, the chosen microcirculatory assessment technique was the cheek pouch preparation. This preparation represents a trustworthy technique since it displays robust, stable blood flow and responsiveness to topic vasoactive drugs. Although the microcirculatory bed observed in the cheek pouch in this investigation was mostly the cutaneous part of it, it is conceivable to consider that it represents systemic alterations. Experiments in humans using laser Doppler and intravital videomicroscopy to examine the skin of the dorsum of the middle finger have shown a good correlation between cutaneous microcirculation and systemic alterations [29]. Microvascular alterations observed on cutaneous tissue attributed to microvascular dysfunction are not only local, but also a systemic process, and studies concerning the cutaneous vascular bed may reproduce alterations on tissues such as muscle [30]–[31].

Enhanced fat depots, features of obese states, incite a unique shift in adipocytes with the installation of an inflammatory and vasoconstrictor profiles. Mature adipocytes secrete an ever-increasing number of growth factors that participate in diverse metabolic processes, particularly insulin resistance. Numberless compounds such as lipoprotein lipase, angiotensinogen, aldosterone, adiponectin, TNF-α and nitric oxide participate in different metabolic and vascular processes, including adipogenesis [32]. The combination of abdominal obesity, impaired glucose and insulin metabolism, dyslipidemia and hypertension is known as metabolic syndrome, an important risk factor for cardiovascular and coronary heart disease mortality. Insulin resistance is a metabolic defect present in this syndrome, associated to type 2 diabetes, obesity, hypertension and dyslipidaemia [33]. In this context, the microcirculation represents an important site to evaluate the profile of adipocytes since systemic inflammation, observed in dysfunctional adipose tissues, can harm the microcirculatory function before perceptible changes on body composition and comorbidities may be observed.

Our findings demonstrated that HF feeding elicited microvascular dysfunction and AET was able to reverse/attenuate most of fat overload damage. Animals from HFEX group presented an important restoration of endothelial function as well as body composition modifications after AET program since these animals did not show any significant changes in either body weight or fat deposition (see in table 2). It is interesting to notice that diet is a necessary component in our study, since microvascular improvements are leashed to reductions on body composition and fat depots stimulated by dietary modifications. Animals that return to standard diet with or without AET presented significantly increased responses to endothelium-dependent vasodilator, indicating an improvement of their endothelial function. Our work showed that there was a significant improvement on HFEX, but more explicit on HFSC and HFSCEX groups concerning vascular reactivity, reflected on vasodilator responses elicited by acetylcholine in all concentrations tested (10^−8^ M, 10^−6^ M and 10^−4^ M - see in figure 3).

Endothelial dysfunction (ED) is characterized by blunted vasodilator responses to a number of stimuli responsible for increasing production or release of endothelial NO such as activation of muscarinic receptors or increase on shear stress by raising blood flow. ED reflects alterations in the endothelial barrier and is consider an early marker of cardiovascular risk that precedes any visible structural atheromatous plaques and traditional cardiovascular risk factors, such as hypertension [34], diabetes mellitus, hypercholesterolemia and obesity [35]. Therefore, studies concerning ED could be applied as therapeutic strategies to prevent these diseases. Fat distribution, in this sense, has been found to be an important determinant of endothelial dysfunction and a strong modulator of microvascular reactivity [36], which reinforces the benefits obtained in the present investigation. High fat diets can be successfully used as inciter of ED, although this model is not only related to ED but also to prostanoid- mediated vasoconstriction in conductance vessels of mice [37] and atherosclerosis in hamsters exposed to high cholesterol diet [38]. Nitric oxide plays an important role on insulin's vasodilator effect as well as in states of insulin resistance, such as obesity and diabetes, characterized by reduced insulin-mediated vasodilatation subsequent to endothelial dysfunction. The mechanisms underlying impaired vascular responses later in life probably involves nitric oxide (NO) and other factors that could interfere with insulin vasodilator effects such as pro-inflammatory adipocytokines like IL-6 and TNF-α [39]–[40].

Ischemia/reperfusion-induced macromolecular permeability increase obtained after 30 min ischemia showed significant reduction on the number of leaks in peak time (10 min) on groups that passed through intervention protocols (HFSC, HFEX and HFSCEX), reflecting improvements on endothelial dysfunction state. Although not significant, there was a higher decrease on leaks in animals exposed to both interventions. Diet and exercise (HFSCEX), showed the highest difference when compared to HF, suggesting that not only the protective effects of exercise, but also the reduction of deleterious effects caused by fat consumption achieved by dietary modification is necessary (HFSC and HFSCEX – see in figure 5). The investigation combining diet changes and moderate aerobic exercise indicates that mediators well known to increase endothelial permeability, such as thrombin, reactive oxygen species, vascular endothelial growth factor (VEGF) and TNF-α, augmented in obesity, could be reduced after exposure to these protocols [41]–[42]. Endothelial properties are affected by mechanical stress and macromolecular permeability increase induced by ischemia/reperfusion demonstrated opposite effects when acute and chronic enhance of shear stress occurred. An acute increase in shear stress promotes an increase of macromolecular permeability count in endothelial cells monolayer, while a chronic increase is responsible for protecting the endothelial monolayer [43], as demonstrated by our findings.

The premise that fat overnutrition after weaning increased body weight and body fat mass could be confirmed in the present investigation. Urogenital, visceral and retroperitoneal fat depots and naso-anal length were greater in HF group at 20th week when compared to HFSC, HFEX and HFSCEX ones. Exercise and dietary implementation proved to be important non-pharmacological ways of treatment, since they were able to decrease body mass after 12 weeks of HF. Data on the literature corroborate our findings since diet-induced obesity in rats chronically subjected to exercise showed that they developed lower levels of adiposity and body mass [44]. Although AET was able to reverse most damages caused by HF feeding, it was not the only effective treatment and dietary modification has proved once again that reduction in caloric intake was essential for changes/improvements not only on body composition but also on microcirculatory dysfunction as seen on modifications observed in groups HFSC and HFSCEX. According to guidelines of the American College of Sports Medicine from 2001 [45] and 2009 [46], changes in caloric intake are necessary to lose and, most importantly, maintain weight. In simplistic terms, when energy intake is reduced below the current needs of the body, weight loss will occur. Obese subjects normally regain their lost weight, showing even worsen body composition scenario after the first weight loss. Therefore, it is necessary not only to enhance the amount of exercise which those subjects are exposed to, but also tailor constantly the diet to each patient needs [Weight management. J. Am Diet Assoc. 2009;109(2):330–46], in order to acquire better responses from exercise. Guidelines point to 150 min.wk^−1^ to 250 min.wk^−1^ to guarantee that lost weight will not be regained [46]. For that purpose, our experimental design matched most guidelines directions, since increased intensity and time of endurance were made, concomitantly with a dietary approach (see AET program and experimental design in figure 1). In order to make a parallel to human intervention it adds an important bias, not usually present in animal experiments, the failure of exercise and dietary interventions in either maintenance and reduction of body weight or fat and to achieve patients' purpose drugs are usually prescribed. These drugs usually act (1) in the central nervous system, decreasing the appetite, (2) reducing the absorption of nutrients or (3) raising body energy expenditure [47]. Their efficiency is related to the capacity of reducing fat depots, maintaining weight loss, diminishing risks related to obesity and ultimately improving life quality. Nowadays the most indicated ones are tesofensin, successor of sibutramine, and orlistat, the first two enhance satiation and thermogenesis [48], and the last one reduces fat absorption through inhibition of gastrointestinal and pancreatic lipases. High cost and serious side effects (psycho-stimulatory, depression, addiction, cardio-excitatory effects, pulmonary hypertension, and valve disease) demand surveillance. Antiobesity agents that possess cardio-excitatory and psycho-stimulatory effects (e.g., phentermine, ephedrine, and caffeine mixture) are still available for the short-term use in some countries and have been abolished in others. To summarize, the rationale of obesity treatment in humans is far more complicated than in animal models.

Several mechanisms are associated to exercise improvements, and some of them are worthwhile mention. Exercise has a close relation to increasing blood flow velocity, modifying laminar shear stress that has an intimate connection to endothelial cell monolayer genetic expression. In fact, blood flow is responsible for the constitutive genetic pattern of endothelial cells [49]. Mechanical agonist stimulus provoked by exercise augments target endothelial genes (e.g. eNOS, MnSOD and NADPH oxidase) and blood flow followed by upregulation of eNOS and Ca^2+^-dependent K^+^ channel 4 (KCNN4) genes [49]. Generally, prolonged unidirectional chronic shear stress elevation is atheroprotective because it increases endothelial anti-inflammatory and antioxidative profiles. The cross-talk between exercise and endothelial cells is important to explain how shear stress acts on the cell surface. Furthermore mechanical alterations of endothelium result in decreased β-adrenergic vascular responsiveness, and consequently attenuated sympathetic outflow [50]. Concomitantly, the skeletal muscle acts as an endocrine organ secreting IL-6, a classical myokine, which increases in an exponential fashion with exercise according to its intensity, duration, recruited mass and endurance capacity. Exercise-derived IL-6 is related to reduction of TNF-α, enhanced s-TNF-r (TNF-α soluble receptor) and IL-10, indicating important anti-inflammatory effects [51] from exercise that could influence the microcirculation. These downstream effects of exercise found in our study could explain the improvements seen in hemodynamic parameters where animals exposed to AET showed a decrease in mean arterial pressure and heart rate.

Adiposopathy reflects a chronic inflammatory state and increased iNOS expression in the aorta of HF group points towards such inflammatory profile. Chronic blockade of NO synthesis in HFD-induced obese mice reduced inflammation and improved insulin signaling to skeletal muscle [52]. Therefore, increased iNOS expression might be a factor associated to endothelial dysfunction in obese groups. It should be mentioned that iNOS expression was not significantly different between groups, probably due to the short intervention time.

Other important marker that may contribute to reduce endothelium-dependent vasodilatation on HF is the lipid profile, strongly associated to an increased risk of cardiovascular disease and atherogenic profile. Nevertheless, studies concerning aerobic training and its effects on lipid profile markers diverge between improvements and neuter consequences, depending on modality and intensity [53]. In our study, no significant differences were found on lipid profile on treated groups, again probably due to the short intervention time. Another factor to be considered was the dietary modification, since those animals that did not passed through calorie reduction (return to normal rodent chow) the damages elicited by high fat exposure could not be completely restored.

Besides the lipid profile, leptin has shown a linear reduction according to the interventions, achieving a significant difference in HFSCEX group, compared to HF. Leptin is produced by fat tissues and it has a clear role in weight control. Obese subjects develop leptin resistance and in our work a significant relationship between plasma leptin and body weight was found. After the exercise protocol there was a linear reduction of these levels, suggesting an increase on leptin sensibility. Our results could be confirmed by data on obese rats subjected to treadmill routine for four weeks [54].

Limitations of our study deal with animal and methodology choices (1) low compatibility to measurement kits, which prevented the evaluation of important adipokines such as TNF-α, MCP-1 and others and (2) the lack of tail, important for blood collection and analysis during exercise exposure. Although attempts to measure blood lactate, pO_2_, pCO_2_ and bicarbonate were made, these failed due to lack of blood drops during the exercise protocol. The last limitation concerned the chosen microcirculatory method, as mention above the cheek pouch present a robust blood flow, clarity and stability, notwithstanding the overriding analyzed tissue, e.g. the window of observation, was the cutaneous one and only few parts of skeletal muscle composed the overall observation, reflecting systemic alterations and improvements.

In summary, we have presented evidence suggesting that the damage elicited on the endothelium by an inflammatory scenario, observed in high fat fed animals, may be attenuated with interventions such as returning to standard chow associated or not to aerobic exercise training, leading to reduction of adipose tissue accumulation and improvement on microvascular reactivity.
