# Correction: Chronic Aerobic Exercise Associated to Dietary Modification Improve Endothelial Function and eNOS Expression in High Fat Fed Hamsters

**DOI:** 10.1371/journal.pone.0111158

**Published:** 2014-10-09

**Authors:** The PLOS ONE Staff

Figure 6 is truncated in the PDF version of the article. Please refer to the XML version for the correctly sized figure. The publisher apologizes for this error that occurred while preparing the manuscript for publication.

The asterisks in Figure 3 and Figure 5 are positioned incorrectly. The authors have provided corrected versions of Figure 3 and Figure 5 here.

**Figure 3:**
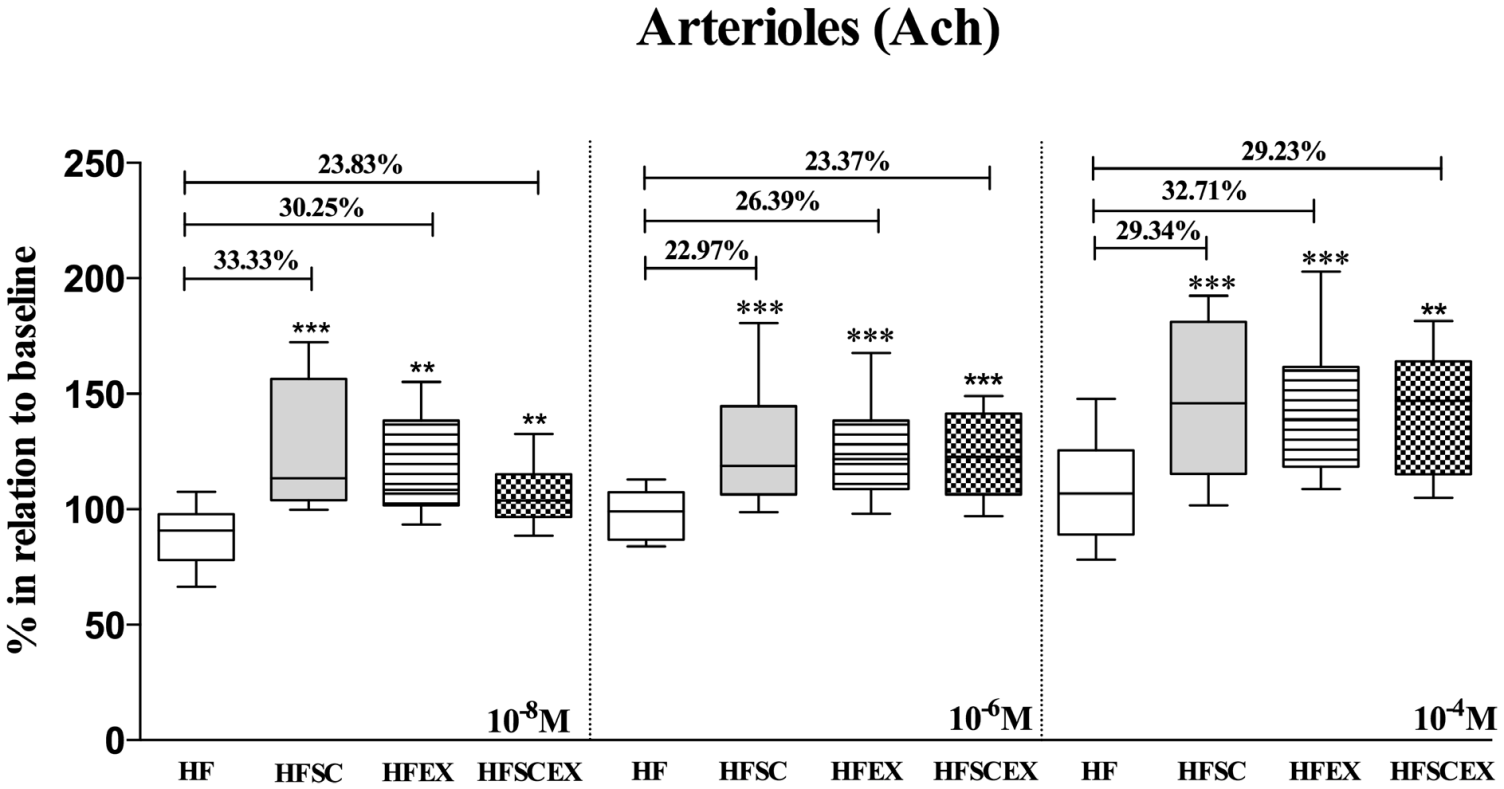
Mean arteriolar diameters after topical application of three concentrations of acetylcholine (10−8, 10−6 and 10−4 M) to the cheek pouch of hamsters fed with high fat diet (HF and HFEX, n  =  10 each) and that had dietary modification associated or not to AET (HFSC and HFSCEX, n  =  10 each) during 20 weeks. Data are shown as changes relative to baseline considered as 100%. Data are expressed as median±10–90 percentile, represented by vertical bars. *Significantly different from sedentary obese control group (p<0.001 and p<0.01, respectively) in all concentrations of Ach.

**Figure 5:**
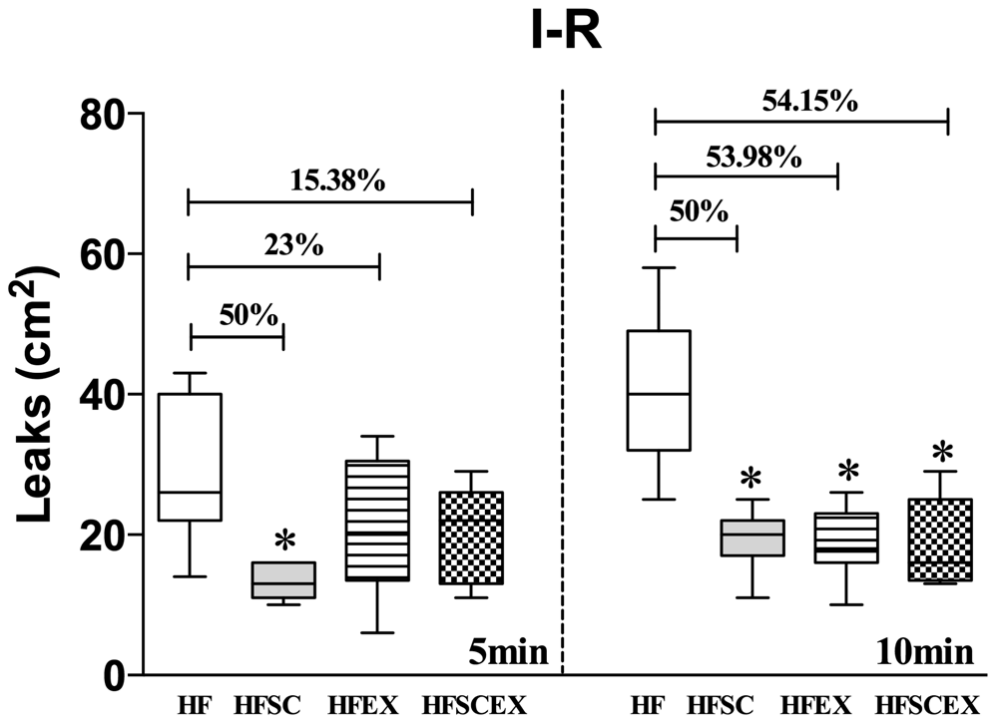
Macromolecular permeability at post capillary venules after 30 minutes ischemia of the cheek pouch of hamsters fed with high fat chow (HF and HFEX, n  =  10 each) and with dietary modification associated or not to AET (HFSC and HFSCEX, n  =  10 each) during 20 weeks. T5, 5 minutes after and T10, 10 minutes after tourniquet release. Data are expressed as median ± 10–90 percentile, represented by vertical bars. * Significantly different from the HF group (p<0.05). HF  =  High Fat Diet. HFSC  =  High Fat Diet + Standard Chow. HFEX  =  High Fat Diet + Exercise. HFSCEX  =  High Fat Diet +Standard Chow/Exercise.
